# The Structural Characterization and Antipathogenic Activities of Quinoin, a Type 1 Ribosome-Inactivating Protein from Quinoa Seeds

**DOI:** 10.3390/ijms22168964

**Published:** 2021-08-20

**Authors:** Sara Ragucci, Daniela Bulgari, Nicola Landi, Rosita Russo, Angela Clemente, Mariangela Valletta, Angela Chambery, Emanuela Gobbi, Franco Faoro, Antimo Di Maro

**Affiliations:** 1 Department of Environmental, Biological and Pharmaceutical Sciences and Technologies (DiSTABiF), University of Campania ‘Luigi Vanvitelli’, Via Vivaldi 43, 81100 Caserta, Italy; sara.ragucci@unicampania.it (S.R.); nicola.landi@unicampania.it (N.L.); rosita.russo@unicampania.i (R.R.); angela.clemente@unicampania.it (A.C.); mariangela.valletta@unicampania.it (M.V.); angela.chambery@unicampania.it (A.C.); 2 Agri-Food and Environmental Microbiology Platform (PiMiAA), Department of Molecular and Translational Medicine, University of Brescia, Viale Europa 11, 25123 Brescia, Italy; daniela.bulgari@unibs.it (D.B.); emanuela.gobbi@unibs.it (E.G.); 3 Department of Agricultural and Environmental Sciences, University of Milan, Via Celoria 2, 20133 Milan, Italy; franco.faoro@unimi.it

**Keywords:** amino acid sequence, *Chenopodium quinoa* Willd, plant pathogens, protein purification, Tobacco Necrosis Virus

## Abstract

Quinoin is a type 1 ribosome-inactivating protein (RIP) we previously isolated from the seeds of pseudocereal quinoa (*Chenopodium quinoa*) and is known as a functional food for its beneficial effects on human health. As the presence of RIPs in edible plants could be potentially risky, here we further characterised biochemically the protein (complete amino acid sequence, homologies/differences with other RIPs and three-dimensional homology modeling) and explored its possible defensive role against pathogens. Quinoin consists of 254 amino acid residues, without cysteinyl residues. As demonstrated by similarities and homology modeling, quinoin preserves the amino acid residues of the active site (Tyr75, Tyr122, Glu177, Arg180, Phe181 and Trp206; quinoin numbering) and the RIP-fold characteristic of RIPs. The polypeptide chain of quinoin contains two N-glycosylation sites at Asn115 and Asp231, the second of which appears to be linked to sugars. Moreover, by comparative MALDI-TOF tryptic peptide mapping, two differently glycosylated forms of quinoin, named pre-quinoin-1 and pre-quinoin-2 (~0.11 mg/100 g and ~0.85 mg/100 g of seeds, respectively) were characterised. Finally, quinoin possesses: (i) strong antiviral activity, both in vitro and in vivo towards Tobacco Necrosis Virus (TNV); (ii) a growth inhibition effect on the bacterial pathogens of plants; and (iii) a slight antifungal effect against two *Cryphonectria parasitica* strains.

## 1. Introduction

Ribosome-inactivating proteins (RIPs) are a group of specific toxins that inhibit protein synthesis [1]. This inhibition is a consequence of ribosome injury due to the enzymatic activity of RIPs, which makes ribosomes unable to interact with transcription factors such as the elongation factor 2 (EF-2, in eukaryotes) or the elongation factor G (EF-G, in prokaryotes), thus blocking the translocation processes [2]. In particular, the enzymatic action of RIPs results in the cleavage of a specific adenine residue (A_4324_ in rat) in a conserved site of the 28S rRNA, known as the Sarcin Ricin Loop (SRL), for which they are classified as rRNA N-glycosylase (EC: 3.2.2.22) [3]. The specific enzymatic action of RIPs promotes cell death by apoptosis through the activation of caspases and caspase-like activities as well as the poly(ADP-ribose) polymerase cleavage [4]. In addition, several RIPs remove adenines from different nucleic acids (poly(A), DNA, RNA and virus RNA), for which these enzymes are described as adenine polynucleotide glycosylases (APG) [5].

RIPs are found in a large number of plants (flowering plants or Angiosperms) [6] and are distributed in different plant tissues (seeds, roots, leaves and bark) [5], although novel findings have highlighted their presence in fungi [7] and metazoan [8], while analogous enzymatic activities (the Shiga-like toxin) have been investigated for a long time in bacteria [9].

RIPs are classified into type 1 or 2 according to the presence or absence of a quaternary structure. Type 1 RIPs are single-chain proteins (~30-kDa with a basic pI) that possess rRNA N-glycosylase activity, while dimeric type 2 RIPs consist of an A-chain (~30-kDa) endowed with enzymatic activity, linked to a B-chain (~34-kDa) with lectin properties and are able to bind oligosaccharides containing galactose moieties [10]. Furthermore, a novel group known as type 3 RIPs has been proposed, with the peculiar structural characteristics of this member (i.e., maize b-32 from maize endosperm and bRIP from barley). RIPs of this new group are synthesised as single domain proenzymes and are subsequently subjected to post-translational changes [11].

To date, the biological role of RIPs is still unknown, although different reports indicate that they may be involved in host defence activities, suggesting that these toxins are functional in conferring a fitness advantage [11]. On the other hand, the interest in these enzymes is due to their potential use for the treatment of several human diseases such as tumours, their employment in the construction of immunotoxins/nanoconstructs to obtain specificity [4,12,13] or in plant biotechnology applications to improve resistance against pathogens [11,14], particularly viruses [15,16].

Some RIPs have also been found in edible plants (e.g., cereals, butternut squash (*Cucurbita moschata*) and bitter melon (*Momordica charantia*)) and are therefore eaten by humans [17,18,19], although their toxicity is reduced or eliminated during digestion or by cooking food at high temperatures. Nevertheless, some type 2 RIPs have been also identified as potential food allergens [19,20].

In this framework, we previously purified and enzymatically characterised a type 1 RIP (named quinoin) from the functional food *Chenopodium quinoa* Willd seeds [21], considering their beneficial action in relation to several human diseases, despite the presence of anti-nutritional factors [22]. Quinoin is a thermostable protein (Tm = 68.2 °C), is cytotoxic against BJ-5ta (human fibroblasts) and HaCaT (human keratinocytes) in a dose- and time-dependent manner and is partially resistant to an in vitro digestive pepsin–trypsin treatment [21]. Although several biochemical aspects of quinoin have been studied, some important questions remain unsolved such as the primary structure characterization and its possible antipathogenic activities.

Taking into account the above, the aim of the present study was: (i) obtain the primary structure of quinoin; (ii) analyse the gene structure of quinoin through a bioinformatics approach considering the genome availability of *C. quinoa*; and (iii) characterise its different activities against fungi, bacteria and plant viruses.

## 2. Results

### 2.1. Protein Purification

The homogenous preparation of quinoin for structural characterization and biological assays was achieved as previously reported [21]. Briefly, a quinoa seed extract was subjected to acid precipitation and soluble proteins were separated, exploiting both the differences in protein size (size exclusion chromatography) and the charge properties (cation exchange chromatography).

**Figure 1:**
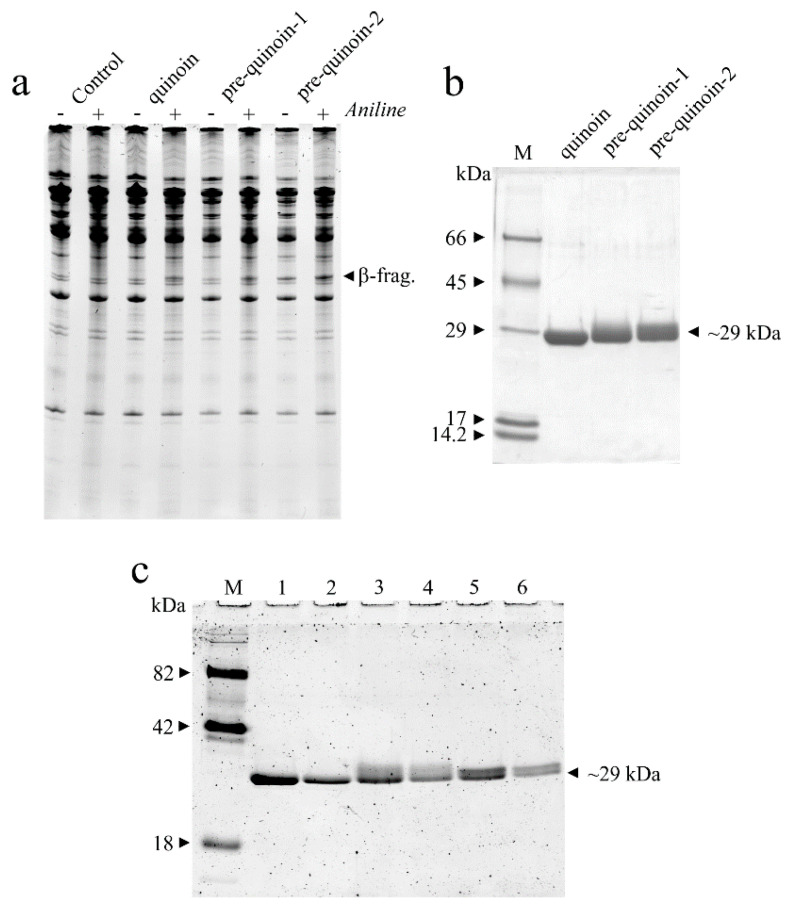
(**a**) rRNA N-glycosylase activity of quinoin, pre-quinoin-1 and pre-quinoin-2 on rabbit ribosomes. rRNA N-glycosylase activity was assayed as indicated in the ‘Material and Methods’ section. Each lane contained 3.0 μg of RNA isolated from either untreated (control) or RIP treated ribosomes from a rabbit. β-frag. indicates the RNA fragment (Endo’s fragment) released as a result of RIP action after acid aniline treatment (+); (−) without aniline treatment. (**b**) Analysis by SDS-PAGE of the three RIPs isolated from *C. quinoa* seeds. M, marker proteins. SDS-PAGE was carried out in 12% polyacrylamide separating gel under reducing conditions. (**c**) A total of 3.0 and 1.5 μg of quinoin (lane 1 and 2, respectively), pre-quinoin-1 (lane 3 and 4, respectively) and pre-quinoin-2 (lane 5 and 6, respectively) were run on 12% SDS-PAGE under reducing conditions, followed by in-gel glycan detection using the Pro-Q Emerald 300 glycoprotein staining kit. Stained proteins were visualised by UV trans-illumination. M, CandyCane™ glycoprotein molecular weight standards.

Moreover, from the last step of purification by CM-Sepharose chromatography in addition to the main protein peak (peak three), which corresponds to quinoin, two minor protein peaks (peak one and peak two) eluted at a lower ionic strength with respect to the quinoin peak, were identified (Figure S1a). The pooled fractions of peaks one and two were able to release the β-fragment diagnostic for RIPs action (N-glycosylase activity), shown in Figure 1a.

In this framework, to obtain a more homogeneous preparation of these novel RIPs, a further purification step by FPLC was carried out for both pools. The Source-S re-chromatography profiles showed the presence of a main principal protein peak, hereafter named pre-quinoin-1 and pre-quinoin-2 (Figure S1b,c). The SDS-PAGE analysis showed that the fractions of pre-quinoin-1 and pre-quinoin-2 contained a single protein band, with a mobility for both corresponding to ~30-kDa (data not shown), for which these protein fractions contained two novel type 1 RIPs. Therefore, the fractions corresponding to pre-quinoin-1 and pre-quinoin-2 were pooled, dialyzed against water and used for further experiments considering their homogeneity shown by the SDS-PAGE analysis (Figure 1b). The yield of the purified proteins pre-quinoin-1 and pre-quinoin-2 was ~0.11 mg/100 g and ~0.85 mg/100 g of seeds, respectively.

Finally, to investigate the possibility that the slight altered migration (smear) in the SDS-PAGE of pre-quinoin-1 and pre-quinoin-2 (see Figure 1b) could be due to glycosylation, a specific analysis for glycoproteins detection was carried out. When analysed by SDS-PAGE and sugar staining, the proteins appeared to be glycosylated (Figure 1c). In particular, glycosylation was more evident for pre-quinoin-1 and pre-quinoin-2 with respect to quinoin, considering the very closed detected band of the latter.

### 2.2. Determination of the Primary Structure of Quinoin

The amino acid sequence of quinoin was obtained by using a strategy based on high-resolution nanoLC-Tandem Mass Spectrometry in order to quickly obtain information on the primary structure of the peptides obtained after tryptic and chymotryptic digestion. This information was then used as a query for mapping the proteins to the *C. quinoa* genome [23] by using the BLASTp algorithm (https://blast.ncbi.nlm.nih.gov; visit date 8 June 2021). Amino acid sequences of both the tryptic and chymotryptic peptides obtained by high-resolution tandem mass spectrometry are reported in Table 1. This survey revealed six proteins that were retrieved as ‘protein synthesis inhibitor-like’ that produced significant alignments. These sequences were: protein synthesis inhibitor PD-S2-like (A.C.: XP_021750597.1), ribosome-inactivating protein PD-L3/PD-L4-like (A.C.: XP_021750694.1), protein synthesis inhibitor PD-S2-like_(2) (A.C.: XP_021737780.1), ribosome-inactivating protein PD-L3/PD-L4-like (A.C.: XP_021726635.1), antiviral protein MAP-like (A.C.: XP_021735486.1) and antiviral protein MAP-like (A.C.: XP_021750669.1). The amino acid sequences of these ‘protein synthesis inhibitors like’ are reported in Figure S2.

**Figure 2:**
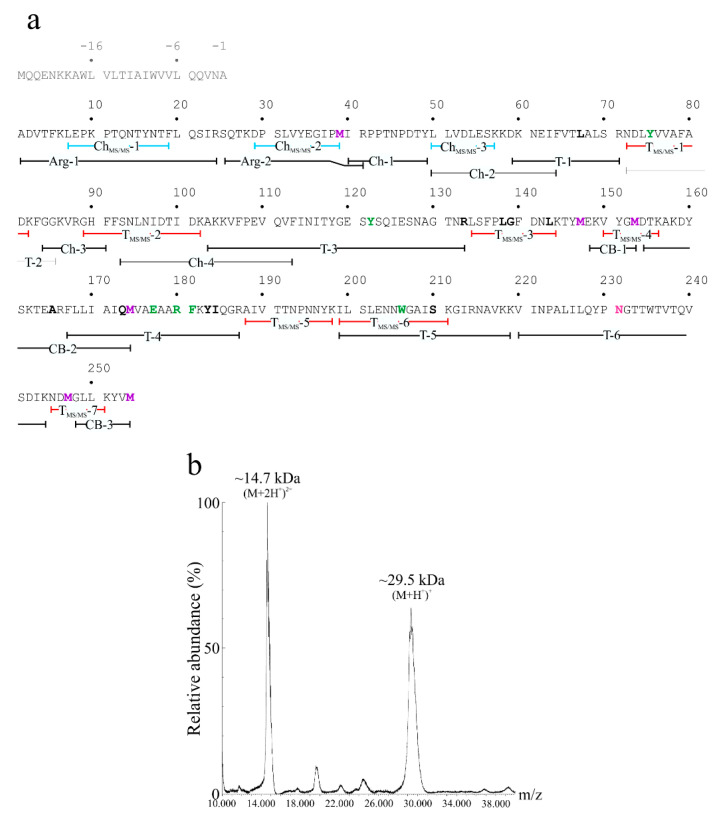
(**a**) Amino acid sequence of quinoin obtained using the protein synthesis inhibitor PD-S2-like A.C.: XP_021750597.1 in the *Chenopodium quinoa* genome as a reference. The overlapping peptides used for assembling the purified protein sequence are reported. Amino acid residues located in the active site and those that might be in contact with the adenine substrate are highlighted in green or bold, respectively [6]. Methionyl residues and glycosylated asparaginyl residue are in magenta and pink, respectively. The N-terminal signal peptide is reported in light grey (position −25 −1). Abbreviations: T_MS/MS_ and Ch_MS/MS_, respectively, tryptic and chymotryptic peptides obtained by high-resolution tandem mass spectrometry. Arg-, Ch-, T- (endoproteinase Arg-C, chymotryptic and tryptic peptides, respectively) and CB (cyanogen bromide fragments) analysed by MALDI-TOF spectrometer. (**b**), MALDI-TOF mass spectrum of native quinoin. The peak at 14.7-kDa corresponds to the doubly charged ion [M+2H^+^]^2+^.

Among these, the protein synthesis inhibitor PD-S2-like (A.C.: XP_021750597.1) exhibited a closer identity with the sequences of both the tryptic and chymotryptic peptides derived from purified quinoin. In this framework, we decided to use the amino acid sequence of the protein synthesis inhibitor PD-S2-like (A.C.: XP_021750597.1) as a reference protein. The sequencing of both the obtained tryptic and chymotryptic peptides provided for ~38% of the amino acid sequence of quinoin (106 out of 279 amino acid residues, referred to the protein synthesis inhibitor PD-S2-like sequence) and their overlap is reported in Figure 2a. Subsequently, for sequence completion and/or verification we decided to perform a comparative peptide mapping by MALDI-TOF mass spectrometry, using also in this case the primary structure of the protein synthesis inhibitor PD-S2-like (A.C.: XP_021750597.1) as a reference protein. To this aim, a new set of peptides derived from endoproteinase Arg-C, chymotrypsin and trypsin as well as the chemical fragmentation (CNBr) were analysed. The accurate M*r* of the peptides or fragments obtained from proteinase digestion and cyanogen bromide cleavage, respectively, were used to assemble the amino acid sequence of quinoin, as reported in Table 2, while the primary structure of quinoin with these overlapping peptides is reported in Figure 2a. In particular, the mapping of position 220–244 was achieved considering the M*r* of two tryptic peptides T-6 and T-6′ (experimental M*r* 3490.28 and 3636.56, respectively), the site of N-glycosylation (Asn231 in the NGT consensus sequence) and the reactivity of quinoin by the glycosylation analysis (see Figure 1c). Indeed, the analyses by the *online* Glycomod tool [24] allowed us to confirm that the M*r*s of 3490.28 and 3636.56 were due to the theoretical molecular mass of the amino acid sequence 220-VINPALILQYPNGTTWTVTQVSDIK-244 (2772.19 Da) plus the molecular masses of the predicted glycan chains: [(Hex)_1_(HexNAc)_2_(Deoxyhexose)_1_] (714.67 Da) for T-6 and [(Hex)_2_ (HexNAc)_2_(Pent)_1_] (862.79 Da) for T-6′. In light of this, the theoretical M*r*s of the glycosylated peptides T-6 and T-6′ were 3487.87 (Δmass = 2.41 with respect to the experimental M*r*) and 3635.99, respectively (Δmass = 0.57 with respect to the experimental M*r*). These predicted possible oligosaccharide structures were retrieved in glycoproteins from plants, such as horseradish peroxidase [25,26].

The strategy allowed the MS mapping of ~99% of the quinoin sequence (251/254 amino acid residues) compared to the protein synthesis inhibitor PD-S2-like (A.C.: XP_021750597.1). Therefore, these data allowed us to confirm that the protein synthesis inhibitor PD-S2-like (A.C.: XP_021750597.1) amino acid sequence is the primary structure of quinoin. In addition, no sequence information was achieved for the N-terminal peptide ‘MQQENKKAWL VLTIAIWVVL QQVNA’ (position −1 −25 in Figure 2a), for which it is most likely the N-terminal signal peptide of quinoin. This evidence was also obtained from the analysis of the protein synthesis inhibitor PD-S2-like amino acid sequence by the SignalP 5.0 server that predicts signal peptides sequences [27]. In particular, this tool predicted a theoretical specific cleavage site between Ala(-1) and Ala1 with a probability of 0.975.

Finally, the quinoin sequence accounted for a calculated molecular mass of 28,766.1 Da, which was in agreement with the value obtained by MALDI-TOF mass spectrometry on the native protein (~29.5 kDa; Figure 2b), taking into account the heterogeneity due to the presence of the glycan chains moiety on Asn231.

Overall, purified quinoin has 254 amino acid residues, one N-glycosylated asparaginyl residue and no cysteinyl residues.

### 2.3. Sequence Comparison, Structural Features and Homology Model of Quinoin

**Figure 3:**
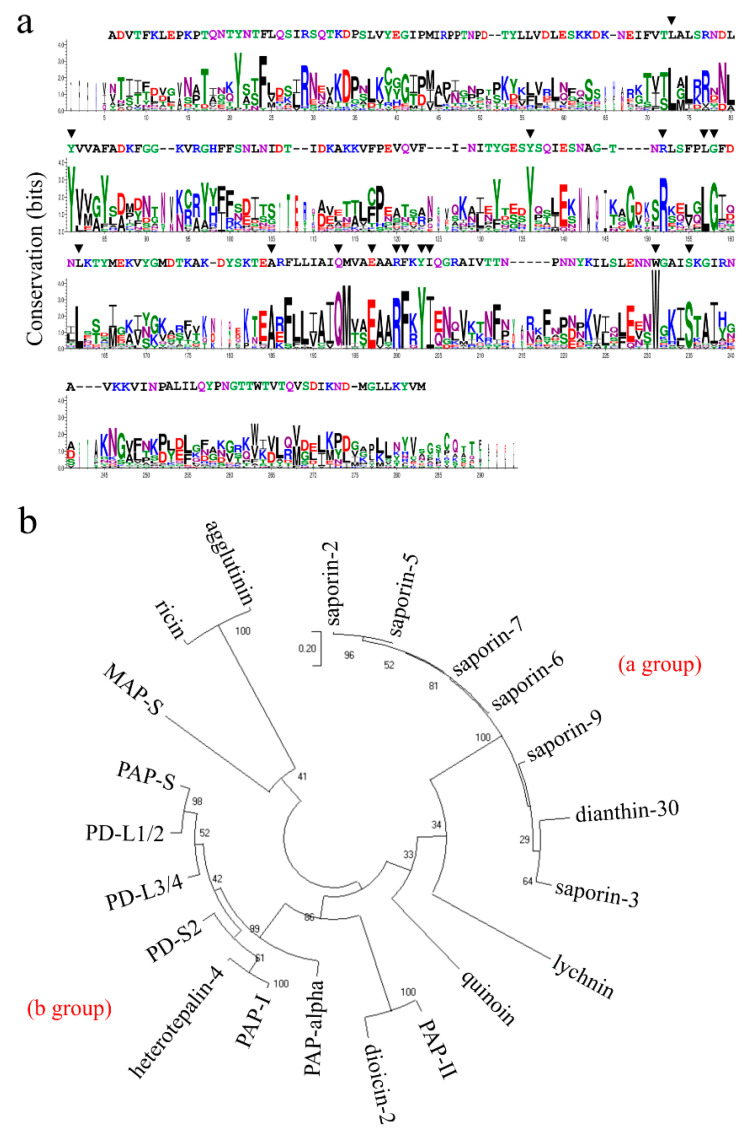
(**a**) Sequence logo between quinoin and ribosome-inactivating proteins. The sequence logo representation of the alignment of sequences (Figure S2) from 20 representative ribosome-inactivating proteins (Table S1) was created as indicated in the ‘Material and Methods’ section. Letter height is proportional to the conservation of that amino acid at that position in the alignment with respect to all the amino acids; letter width is proportional to the conservation of that amino acid but includes gaps. The colour of each amino acid is according to its chemical properties: polar (G, S, T, Y, C), green; neutral (Q, N), purple; basic (K, R, H), blue; acidic (D, E), red; hydrophobic (A, V, L, I, P, W, F, M), black. The sequence of quinoin is indicated above the logo. ▼ indicates the conserved amino acid residues in the alignment. (**b**) Molecular phylogenetic analysis by the Maximum Likelihood method of quinoin and 20 representative ribosome-inactivating proteins. The evolutionary history was inferred as indicated in the ‘Material and Methods’ section. The name of the ribosome-inactivating proteins is indicated, while the species and the accession numbers are reported in Table S1. All the sequences were retrieved and processed as indicated in the ‘Material and Methods’ section.

In order to obtain information regarding the identity/similarity of quinoin with other RIPs and to gain insight into its structural features, we performed a search for sequence homology. Among the numerous sequences showing a degree of identity, ranging from about 50% to about 30%, we focused our interest on those reported in Table S1. In Figure S3, the quinoin sequence is aligned with those of a number of type 1 RIPs found in the Caryophyllales order (*Phytolacca americana*, *Phytolacca dioica*, *Saponaria officinalis*, *Dianthus caryophyllus*, *Silene chalcedonica* and *Mirabilis jalapa*) and two A-chain of type 2 RIPs found in *Ricinus communis*. The multiple alignment analysis showed that, in all the RIPs evaluated, the active site residues (Tyr75, Tyr122, Glu177, Arg180, Phe181 and Trp206; numbering refers to the quinoin amino acid sequence) involved in the enzymatic mechanism (Glu177 and Arg180) and in the binding with adenine (Tyr75, Tyr122, Phe181 and Trp206) were conserved [6]. Furthermore, considering the obtained logo from the alignment performed (Figure 3a), other amino acid residues were conserved (i.e., Leu67, Arg133, Leu138, Gly139, Leu143, Ala165, Gln173, Tyr183, Ile184 and Ser210 numbering refers to the quinoin amino acid sequence), which correspond to the positions 73, 152, 157, 158, 162, 185, 193, 203, 204 and 235 in the sequence logo. Although many of these residues do not participate directly in the catalytic site, they are important for the correct rearrangement of the fold of the RIPs, considering the organization necessary for the recognition of the target adenine (active-site cleft) in the SRL loop and to expand the network able to promote catalysis by the internal dynamics of these enzymes [6,28]. In particular, experimental data reported in the literature shows that: (i) Ser235 (Ser210 in quinoin) has an important role in stabilizing the conformation of Trp231 (Trp206 in quinoin) side chain through the formation of a specific hydrogen bond [29]; (ii) while Arg152 (Arg133 in quinoin) is an amino acid residue that participates in the binding to the SRL loop [30].

Phylogenetic relationships were investigated among the 21 RIPs sequences analysed by using the Maximum Likelihood method. The sequence of both ricin and agglutinin A-chains, two ribosome-inactivating proteins from *R. communis* belonging to the Euphorbiales order, were used as an outgroup. Two main groups were highlighted by the analysis of the unrooted phylogenetic tree (Figure 3b). The first group (a) included quinoin, lychnin (*S. chalcedonica*), dianthin-30 (*D. caryophyllus*) and the saporins (*S. officinalis*), while all other RIPs (PAP-S, PD-L1/2, PD-L3/4, PD-S2, heterotepalin-4, PAP-I, PAP-α, diocin-2 and PAP-II) were located on a separate branch group (b) all belonging to the *Phytolaccaceae* family. These findings suggest that quinoin shares common ancestors with lychnin, dianthin-30 and saporins. It is interesting to note from a structural point of view that all the proteins that belonged to group (b) (*Phytolaccaceae*) had four cysteinyl residues, while quinoin and other members of group (a) were without cysteinyl residues (except for lychnin, two cysteinyl residues), as shown in Figure S3. This could indicate a different selection pressure and distinct functional roles in these plants. These divergent structural features between quinoin and type 1 RIPs were confirmed by testing the possible cross-reactivity between quinoin and several type 1 RIPs from *P. dioica* (PD-S2, type 1 RIPs from the seed of *P. dioica* [31]; PD-L1 and PD-L4, type 1 RIPs from the leaves of *P. dioica* [32]) using both anti-quinoin and anti-PD-S2 polyclonal rabbit antibodies, respectively. The western blot analysis reported in Figure S4 highlighted a very weak cross-reactivity among quinoin and PD-S2, PD-L1 and PD-L4, confirming the different structural properties that exist between quinoin and type 1 RIPs from *Phytolaccaceae*.

**Figure 4:**
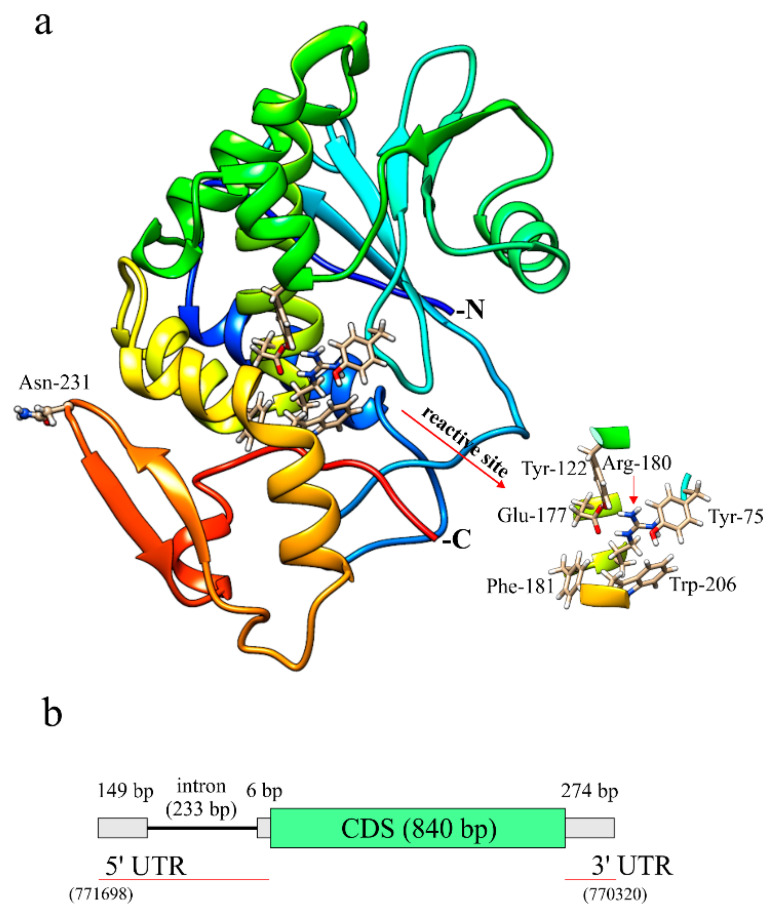
(**a**), Three-dimensional molecular model obtained for quinoin. In this model, reported as a backbone ribbon, the secondary structure topology is shown. N- and C-, indicate N- and C-terminus amino acid residues of the protein, respectively. In the insert, we report the six amino acid residues present in the catalytic site, common to all RIPs [6]. (**b**) Schematic structure of the quinoin gene region with a single intron at 5′UTR. This information is derived from the study on the NCBI reference sequence NW_018745569.1.

Finally, in order to carry out a predictive study of the structure of quinoin, we used the online I-TASSER service; the program built a three-dimensional model of quinoin by selecting (automatically) the three-dimensional structure of RIPs as a template. For quinoin, the computational modeling calculation generated a single conformer with a C-score of 1.09 indicating the good quality of the predicted three-dimensional structure. The overall fold of the quinoin model, including the secondary structure elements, is shown in Figure 4a. The three-dimensional structural model indicated that quinoin presents a typical ‘RIP-fold’ consisting of two major domains: an N-terminal domain with β-strands and α-helices and a C-terminal containing predominantly α-helices [33]. Moreover, it is evident that the amino acid residues that make up the active site of quinoin are present in the same cleft (insert of Figure 4a).

From the three-dimensional structural model of quinoin, the position of N-glycosylated Asn231 was also investigated. This asparaginyl residue is part of an evident β-hairpin in the C-terminal region (position 225–237) that, as has been reported by many authors, plays a role in biological activity of RIPs. Indeed, several studies have reported that changes in this C-terminal region can alter: (i) the interaction with membrane models [34]; (ii) the RNA N-glycosylase activity and the adenines released from supercoiled DNA [35]; and (iii) the antiviral activity or some other biological action of these enzymes [36,37]. Considering as above, the N-glycosylation of Asp231 could have a regulatory role in quinoin.

### 2.4. Gene Organization of Quinoin

The pioneering work of Stirpe et al. reported that the increase in the inhibitory activity of protein synthesis and activity on DNA is due to the presence of RIPs in the leaves of both *Hura crepitans* L. and *P. americana* when subjected to heat or osmotic stress [38]. Since then, experimental evidence has confirmed the control of the expression of RIPs at the gene level during abiotic and biotic stress in plants [39,40]. The de novo assembly of the pokeweed (*P. americana*) genome showed that the genes of both the pokeweed antiviral protein (PAP) and the PAP isoforms exhibited a long 5′ UTR containing one intron and cis-regulatory elements associated with diverse biotic and abiotic stresses [41].

In this framework, with information on the genome of *C. quinoa*, [23] and, in particular, on the genomic sequence coding for quinoin (A.C.: NW_018745569.1 in Gene Bank), we decided to investigate this region through a bioinformatics approach. The gene model coding for quinoin is shown in Figure 4b. The open reading frame (ORF) of quinoin was annotated as a single exon of 840 bp (Figure S5), while the gene model revealed an intron within the 5′ UTR (~233 bp). This model was similar to the gene models retrieved for the PAP isoforms. Therefore, the gene organization suggested that quinoin protein expression could be subjected to particular gene regulation in biotic and abiotic stresses given the presence of the 5′ UTR region of both the intron and the leader intron [41].

### 2.5. Comparative MALDI-TOF Mass Spectrometry Mapping of Pre-Quinoin-1 and Pre-Quinoin-2

In order to acquire the possible structural differences between quinoin and both pre-quinoin-1 and pre-quinoin-2 in addition to glycosylation, we decided to perform a comparative tryptic peptide mapping, using the quinoin primary structure as a reference peptide profile. Thus, the two proteins were first digested with trypsin and then the mixture of peptides was analysed by MALDI-TOF mass spectrometry.

The M*r*s of the tryptic peptides retrieved for the digested pre-quinoin-1 and pre-quinoin-2 forms are reported in Table 3. A coverage of about 75% and 41% for pre-quinoin-1 and pre-quinoin-2, respectively, was achieved considering the theoretical M*r* of the tryptic peptides obtained from the amino acid sequence of quinoin.

Therefore, taking into account the MALDI-TOF-MS peptide mapping, it is important to consider that both pre-quinoin-1 and pre-quinoin-2 type 1 RIPs are different glycosylated forms of quinoin, the major glycoform isolated from *C. quinoa* seeds. Moreover, a thorough analysis of the theoretical glycosylation sites in the quinoin primary structure revealed a second glycosylation site (Asn115 in the NIT consensus sequence), which could justify the presence of these glycoforms. This evidence is in agreement with the different glycosylated glycoforms of PD-S2, the major type 1 RIP characterised in the seeds of *P. dioica* [31].

### 2.6. Antiviral Activity of Quinoin on TNV and TMV

Several type 1 RIPs are known for their antiviral properties against many virus pathogens for plants, animals and bacteria. In light of this, several authors have proposed these enzymes as possible candidates to combat infectious diseases and, in particular, to control crop virus diseases, also considering the lack of specific antiviral products used in the open field [15].

**Figure 5:**
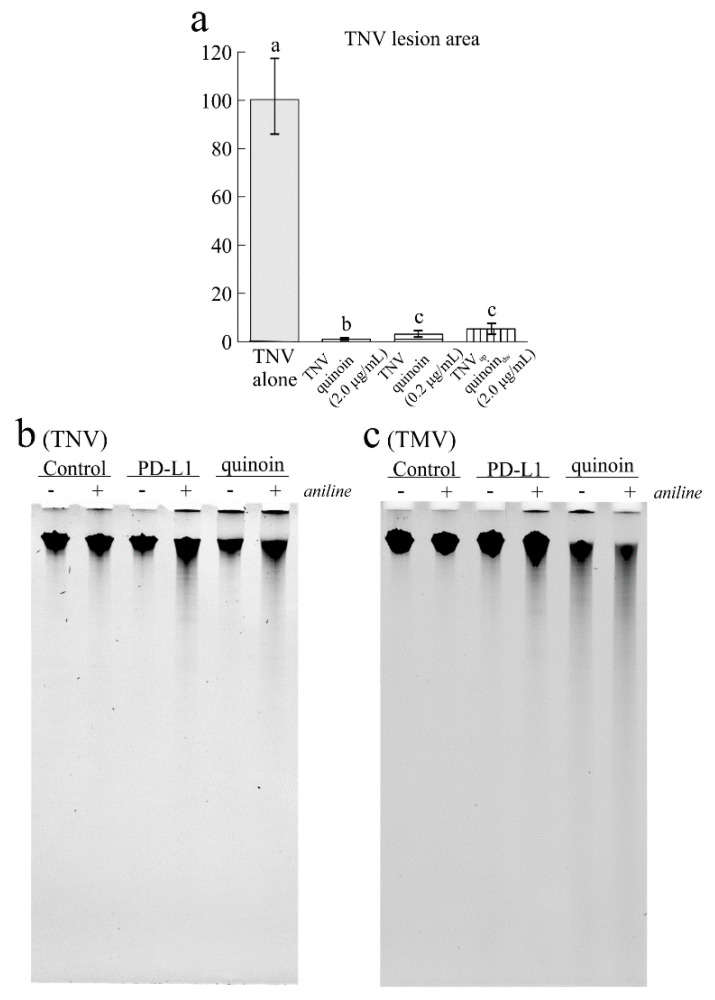
(**a**) The area of TNV lesions developed on bean cotyledonary leaves when the virus was inoculated alone or mixed with different concentrations of quinoin on the adaxial (up) leaf surface, or when inoculated separately from quinoin. The latter was applied on the abaxial leaf surface (down). Data are expressed as percentages of the total lesion area compared to the control (TNV alone) and refer to three independent experiments; different letters represent significant differences according to Fisher’s least significant difference test at *p* < 0.05; the same letter indicated no significant difference. (**b**,**c**) The adenine polynucleotide glycosylase activity of 3.0 μg of quinoin, which was assayed on both TNV and TMV viral RNAs as indicated in the ‘Material and Methods’ section. Each lane contained 3.0 µg of RNA. Samples were treated (+) or not (−) with acid aniline.

Thus, we investigated the possible antiviral action of quinoin against TNV, which affects numerous cultivated plants. In a preliminary experiment, quinoin was tested at 2.0 and 10 µg/mL (70 and 350 nM), concentrations that had previously been shown to be effective in the case of PD-L1 and PD-L4, two type 1 RIPs from the leaves of *P. dioica* [16]. At both concentrations, quinoin completely abolished the TNV infection when inoculated together with the virus suspension in the same leaf surface (data not shown). On the basis of this result, quinoin was tested at 2.0 and 0.2 µg/mL (70 and 7 nM) in three independent experiments which showed that the most diluted solution was able to greatly reduce the virus infection (−96.88%) when mixed with the virus inoculum (Figure 5a and Figure S6). Again, the concentration of 2.0 µg/mL almost completely abolished the infection as assessed by the very few and small lesions (−99.99 total infected area) in respect to the plants inoculated only with the virus suspension in water (Figure 5a and Figure S6).

Interestingly, quinoin was able to inhibit the virus infection when it was also applied on the abaxial leaf surface separately from the TNV that was inoculated on the adaxial surface. In this case, the reduction in the necrotic lesions with 2.0 µg/mL of quinoin was still very high (−94.72%) suggesting a synergistic action of this RIP against both viral and ribosomal RNA. Indeed, this evidence shows that there is no need for direct contact between virus and toxin; the virus inhibition would mainly occur inside the plant cells at the onset of infection [16]. In this framework, we investigated the potential effects of quinoin directly on the genomic RNA of TNV. As displayed in Figure 5b, quinoin promoted the in vitro depurination of the genomic RNA of TNV, which, upon treatment with acid aniline, led to the partial degradation of the polyphosphate RNA backbone (smear after aniline treatment). Finally, in order to confirm the in vitro depurination of the RNAs of the genomic virus plant, the same experiment was carried out on TMV genomic RNA, proving again the effect of quinoin against TMV RNA, as shown by gel electrophoresis (Figure 5c). Overall, these data highlight the depurination capacity of type 1 RIPs on genomic virus plant RNAs as previously reported for type 1 RIPs from *P. dioica* and *Beta vulgaris* L. [37,42].

### 2.7. Antimicrobial Activity

RIPs are a class of defence proteins that could act as antimicrobial agents. Indeed, their antipathogenic activities have been reported against both bacteria and fungi, which makes them active subjects in plant defence [11,17,43].

In light of this, we tested the antibacterial activity of quinoin towards some human and plant pathogenic bacteria. Quinoin showed a growth inhibition effect on the plant pathogens *P. syringae* pv. *phaseolicola* and *P. syringae* pv. *actinidiae* (Table 4). This activity was dose-dependent and showed the maximum inhibition at 100 µg/mL. On the other hand, no effect was observed on the human pathogens *S. aureus* and *S. enterica* subsp. *enterica serovar enteridis*.

The antifungal activity of quinoin was tested on two strains of *Cryphonectria parasitica*, a parasitic fungus of chestnut trees. In this case, no inhibition halo was observed in either strain (Table 4) even though a slight effect on fungal growth was observed for both the *C. parasitica* strain E4 and strain E13 (Figure S7). In this framework, these data confirm that quinoin possesses antifungal activity although to a lesser extent, as reported for other RIPs against hyphomycete fungi such as *Verticillium dahlia*, *Alternaria solani*, *Fusarium oxysporum solani* [43,44,45] and other fungal genera [11].

## 3. Discussion

Quinoin is a type 1 RIP found in *C. quinoa* (quinoa) seeds and exhibits toxicity against different cell lines in vitro. In this framework, this toxin represents a possible anti-nutritional factor in quinoa seeds besides the presence of saponins, phytic acid, tannins, oxalates and trypsin inhibitors, commonly known as anti-nutritional factors [22]. Indeed, several studies have reported that some consumers are affected by innate immune responses or an insurgence of allergies or intolerances [46,47]. In this context, increased information on this type 1 RIP is of interest to verify the real impact of this toxin in a food that is highly requested today.

Thus, in this work, the amino acid sequence of quinoin was determined by using a combined approach based on *C. quinoa* genome analysis and mass spectrometry. This toxin is a glycoprotein and its polypeptide chain consists of 254 amino acid residues, without cysteinyl residues. Similarities in the protein database have been found with other known RIPs and a predictive three-dimensional structure model obtained by homology modeling showed a typical RIP-fold [33]. In addition, an identical spatial rearrangement of amino acid residues forming the active site pocket was retrieved [6]. Through a bioinformatics approach, it is interesting to note that the organization of the genomic sequence coding for quinoin showed a single exon containing ORF and an intron within the 5′ UTR. This organization is similar to the gene models retrieved for PAP isoforms, type 1 RIPs from *P. americana*, and seems to be necessary in gene regulation during biotic and abiotic stresses [41]. Moreover, two glycoproteins (electrophoretic migration of ~30-kDa), able to release the β-fragment, named pre-quinoin-1 and pre-quinoin-2, were purified and identified as different glycosylated forms of quinoin.

Finally, the antipathogenic activities of quinoin against several types of pathogens (fungi, bacteria and plant viruses) were evaluated. Quinoin possesses a strong antiviral activity towards TNV, even stronger than other previously tested RIPs. Interestingly, our data showed that this antiviral activity likely depends on both protein synthesis inhibition (N-glycosylase activity against ribosomes in cells) and depurination action on RNA virus genomes (activity verified in vitro on both TNV and TMV RNAs). In addition, this toxin showed a growth inhibitory effect on plant pathogen bacteria and a slight effect when tested against two *C. parasitica* strains.

Overall, this investigation improves the knowledge of the structural features and the different antipathogenic activities of quinoin. In particular, the antipathogenic activities of this toxin could be used for tuning its possible use as a biotechnological tool.

## 4. Materials and Methods

### 4.1. Materials

Chemicals for protein purification chromatography were obtained as previously reported [48,49]. Other chemicals were from Merck Life Science (Milan, Italy). HPLC-grade solvents and reagents were obtained from VWR International (Milan, Italy). Cyanogen bromide was obtained from Fluka (Milan, Italy). Trypsin, chymotrypsin and endoproteinase Arg-C sequencing grades were obtained from Merck Life Science.

### 4.2. Purification of Type 1 RIPs from the Seeds of C. quinoa

Quinoin and minor forms of type 1 RIPs were purified according to the procedure published by Landi et al. (2021). In addition, for the purification of minority forms, after the last chromatography step on a CM-Sepharose column equilibrated in 5.0 mM Na/phosphate, pH 7.2 (buffer A) and eluted in the same buffer with a NaCl (buffer B) gradient up to 0.15 M (reservoir A, 500 mL, reservoir B, 500 mL; total volume 1 L), a further purification step was introduced. In particular, the minor forms were re-chromatographed by FPLC on an AKTA Purifier System (Amersham Pharmacia; Milan, Italy), using a Source 15S PE 4.6/100 column, equilibrated in buffer A and eluted applying a linear gradient from 0 to 50% of buffer A containing NaCl 0.3 M over 60 min (flow rate 1.0 mL/min).

### 4.3. Enzymatic Assays

The depurination assay (rRNA N-glycosylase assay) was conducted as previously described [37] on rabbit reticulocytes lysates as the substrate.

### 4.4. Analytical Procedures

Quinoin homogeneity was determined by SDS-PAGE with a Mini-Protean II (Bio-Rad; Milan, Italy) using a 6% stacking and 15% separating polyacrylamide gel under reducing conditions; a precision plus protein kit (Bio-Rad) was used as reference proteins. Protein concentration was determined by a Pierce BCA Protein Assay Kit (Life Technologies Italia Fil., Monza, Italy). Protein desalting was obtained by a reversed-phase HPLC (RP-HPLC) on a C-4 column (4.6 × 250 mm, 5.0-µm particle size, 300 Å; Phenomenex, Castel Maggiore, Bologna, Italy) as previously reported [50]. The glycosylation analysis was performed in gel after SDS-PAGE by using the PRO-Q EMERALD 300 GLYCOPROT PROBES KOMBO (Life Technologies Italia). In other to investigate the cross-reactivity of RIPs, western blot protein samples were separated by 15% SDS-PAGE and then transferred onto a PVDF membrane (Millipore Corp. for western blot) [48]. The primary polyclonal rabbit antibodies used (dilution 1:3000) were anti-quinoin or anti-PD-S2 (Bio-Fab research, Rome, Italy). Proteins were visualised by enhanced chemiluminescence (ECL, GE Healthcare, Chicago, IL, USA) using a ChemiDocTM XRS system and analysed by Quantity One software (date of use 12.6.2021; Bio-Rad, Milan, Italy).

### 4.5. Digestion and Chemical Fragmentation of Quinoin

The chemical fragmentation of quinoin (50 µg) with cyanogen bromide (CNBr) was carried out in 70% TFA as a solvent as previously described [50]. Enzymatic hydrolyses of quinoin (50 µg) were achieved by dissolving the protein in 50 mM (NH_4_)HCO_3_ containing 10% acetonitrile. Subsequently, the protein was incubated with TPCK-treated trypsin, chymotrypsin or endoproteinase Arg-C with an enzyme/substrate 1:50 (*w*/*w*) final ratio added in three steps (the first at 0 h (E/S: 1:200); the second at 4 h (E/S: 1:100) and the third at 12 h (overnight, E/S: 1:50); 16 h total). Mixtures were then incubated at 37 °C, acidified (pH < 3.0) and frozen for subsequent analyses [50,51]. The Trypsin digestion of minor forms of RIPs isolated in *C. quinoa* seeds was carried out as reported above for quinoin.

### 4.6. High Resolution NanoLC-Tandem and MALDI-TOF MS Analyses

Mass spectrometry analyses on quinoin tryptic or chymotryptic digests (50 fmol) were performed on a Q-Exactive Orbitrap mass spectrometer equipped with an EASY-Spray nanoelectrospray ion source (Thermo Fisher Scientific, Bremen, Germany) and coupled to a Thermo Scientific Dionex UltiMate 3000RSLC nano-system (Thermo Fisher Scientific, Monza, Italy). The solvent composition was 0.1% formic acid (FA) in water (solvent A) and 0.1% FA in acetonitrile (solvent B). Peptides were loaded on a trapping PepMapTM100 μCartridge column C-18 (300-μm × 0.5 cm, 5.0-μm, 100 Å) and desalted with solvent A for 3 min with at a flow rate of 10 μL/min. After trapping, eluted peptides were separated on an EASY-Spray analytical column (50 cm × 75 μm ID PepMap RSLC C-18, 3 μm, 100 Å). The run condition involved heating to 35 °C and a flow rate of 300 nL/min. The gradient for the peptides separation was 4% B for 3 min, from 4 to 55% B in 60 min, from 55 to 70% B in 10 min, and from 70 to 95% B in 2 min. Eluting peptides were analysed on the Q-Exactive mass spectrometer operating in positive polarity mode set-up as reported in previous works [52].

The relative molecular mass (M*r*) of desalted quinoin together with the peptides from the enzymatic and chemical hydrolyses were determined by using a MALDI-TOF mass spectrometer (Waters Micromass Co., Manchester, UK) as previously reported [51,52].

The Glycomod tool was used to predict theoretical glycosidic chains (https://web.expasy.org/glycomod/; visit date 8 June 2021).

### 4.7. Sequence Analysis and Homology Modeling

RIPs similar to quinoin were obtained from the Uniprot database (http://www.uniprot.org/; visit date 8 June 2021) by using BLAST (https://www.uniprot.org/blast/; visit date 8 June 2021). Only reviewed sequences (records manually annotated and with information extracted from the literature and curator-evaluated computational analysis) were used for subsequent analyses. The signal peptide, connecting peptide and processed peptide sequences were removed in order to use only mature protein sequences. The sequence alignment was performed using the ClustalW tool available online (https://embnet.vital-it.ch/software/ClustalW.html; visit date 8 June 2021). The similarity/identity matrix was obtained using the Sequence Identity and Similarity (SIAS) tool (http://imed.med.ucm.es/Tools/sias.html; visit date 8 June 2021). The sequence logo was obtained using WebLogo tool available online (https://weblogo.berkeley.edu; visit date 8 June 2021). The evolutionary history was performed by using MEGA 10.0.5 (Molecular Evolutionary Genetics Analysis; https://www.megasoftware.net/ (visit date 8 June 2021) [53]. For this procedure, the Maximum Likelihood method based on the JTT matrix-based model option was used. Phylogeny was tested by 1000 bootstrap replications.

A model of the three-dimensional structure of quinoin was obtained using the automated I-TASSER service available at the site https://zhanglab.ccmb.med.umich.edu/I-TASSER/ (visit date 8 June 2021). The selection of templates was performed by using the I-TASSER that at first identifies structure templates by LOMETS from the PDB library. The online procedure yielded the three-dimensional model on the basis of the multiple-threading alignments by LOMETS and the iterative TASSER simulations [54]. The predicted three-dimensional structure of the protein was visualised and analysed using the software Chimera [55].

The study of the genomic sequence of the quinoin gene was performed by using the tools available on GenBank (https://www.ncbi.nlm.nih.gov/genbank/; visit date 8 June 2021). The genomic sequence for NW_018745569.1 was obtained from *C. quinoa* cultivar QQ74.

### 4.8. Plant Material and Viruses for Bioassays

*Nicotiana tabacum*, cv. White Burley, *N. benthamiana* and *Phaseolus vulgaris*, cv. Borlotto Nano Lingua di Fuoco, grown in a greenhouse at 24 ± 2 °C, RH 60 ± 5%, 16 h/8 h light/dark period, were used in this study.

The Tobacco Mosaic Virus (TMV) was propagated in *N. tabacum* and purified from these plants following the Gooding and Hebert protocol [56]. The Tobacco Necrosis Virus (TNV) was propagated on *N. benthamiana* and purified from the systemic infected plants following Montalbini and Polverari protocol [57].

### 4.9. Evaluation of the Antiviral Activity of Quinoin In Vivo on TNV

Fully expanded cotyledonary leaves of *P. vulgaris*, cv. Borlotto Nano Lingua di Fuoco, were inoculated on the adaxial surface by rubbing them with a mixture of quinoin at different concentrations (10 to 0.2 µg/mL) and purified TNV (1:1), using a 600-mesh carborundum as an abrasive. The final virus concentration in the inoculum was 100 ng/mL. In some other experiments, quinoin was applied on the abaxial leaf surface while the virus was inoculated on the adaxial one in order to avoid contact between them. As controls, some plants were inoculated respectively with TNV alone, or with quinoin at different concentrations or water. Plants were observed for the development of lesions for up to 5 days. When the lesions were fully developed, the infected leaves were detached and immediately scanned at a 300 DPI resolution. Images were analysed with Global Lab (Data Translation, Norton, MA, USA) to determine the necrotic lesion area. The quinoin inhibitory activity was calculated as a percentage of the reduction in this area in respect to the leaves only inoculated with TNV. In total, 24 leaves from three independent experiments were analysed for each treatment.

### 4.10. Adenine Polynucleotide Glycosylase Activity of Quinoin on the RNA of TNV and TMV In Vitro

The N-glycosylase activity of RIPs was assayed in 100 μL samples containing 5.0 μg of TNV or TMV RNA, which were incubated with 3.0 μg of quinoin for 1 h at 37 °C. After treatment, the RNA was analysed by phenolization, treated with 1.0 M aniline acetate (pH 4.5), precipitated with ethanol and separated by gel electrophoresis (15 mA for 1 h) and visualised by ethidium bromide staining [37].

### 4.11. Evaluation of the Antibacterial Activity of Quinoin

The agar diffusion assay was carried out using two different human pathogenic species of Gram-negative and positive bacterial strains and two Gram-plant pathogenic strains. Gram-negative *Salmonella enterica* subsp. *enterica serovar enteridis* ATCC 13,076 and Gram-positive *Staphylococcus aureus*, *Pseudomonas syringae* pv. *phaseolicola* and *Pseudomonas syringae* pv. *actinidiae*. A single colony of each strain was inoculated with 10 mL of Luria Broth (LB, Merck, Darmstadt, Germany) and incubated overnight at 37 and 30 °C for human and plant pathogens, respectively. A total of 100 µL of the bacterial culture was spread over LB agar plates. To check the antimicrobial activity of quinoin, 10 μL of the different diluted solution (20, 50 and 100 µg/mL) were loaded on to 4 mm filter paper discs and placed on the inoculated plates. For reference, 10 µL of MilliQ water was added on the paper dish in the same inoculated plates. Plates were incubated for 18 h at 37 and 30 °C for human and plant pathogens, respectively. The inhibition zone was visualised by crystal violet staining as previously described [58], with some modifications. The area with no growth of bacteria around the dish was measured using a ruler.

### 4.12. Evaluation of the Antifungal Activity of Quinoin

The antifungal activity of quinoin was evaluated from two *Cryphonectria parasitica* strains (E4 and E13) deposited in the collection of the Agri-food and Environmental Microbiology Platform (PiMiAA), University of Brescia, Italy. The radial growth inhibition assay was carried out as previously described [43]. Briefly, a mycelium plug was placed in the centre of a Potato Dextrose Agar plate and a dish with different amounts of quinoin (0.4, 0.8 and 2.1 mg/mL) or water at the border. Antifungal activity was observed as a crescent-shaped zone of inhibition at the mycelial front. The effect on fungal growth was expressed qualitatively, according to the procedure of Schlumbaum et al. (1986) [59].

## Figures and Tables

**Table 1:** Amino acid sequences of both tryptic (A) and chymotryptic (B) peptides from quinoin obtained by high-resolution nanoLC-Tandem Mass Spectrometry. Sequence, number of missed cleavages sites (MC), experimental masses of precursor ions, charge state and theoretical molecular weights of peptides (MH^+^) together with mass accuracies are reported.

Sequence	MC	Charge	*m*/*z* [Da]	MH^+^ [Da]	Δmass [ppm]	Sequence Position	Reported in Figure 2
A							
[R].NDLYVVAFADK.[F]	-	2	627.822	1254.636	0.45	72–82	T_MS/MS_-1
[R].GHFFSNLNIDTIDK.[A]	-	3	540.938	1620.801	0.59	89–102	T_MS/MS_-2
[R].LSFPLGFDNLK.[T]	-	2	625.842	1250.677	0.72	134–144	T_MS/MS_-3
[K].VYGMDTK.[A]	-	2	407.194	829.381	0.56	150–156	T_MS/MS_-4
[R].AIVTTNPNNYK.[I]	-	2	617.824	1234.64	0.59	188–198	T_MS/MS_-5
[K].ILSLENNWGAISK.[G]	-	2	772.892	1444.78	1.01	199–211	T_MS/MS_-6
[K].NDMGLLK.[Y]	-	2	395.710	790.412	0.44	245–251	T_MS/MS_-7
B							
[K].LEPKPTQNTY.[N]	-	2	595.806	1190.604	0.64	7–16	n.r.
[K].LEPKPTQNTYNTF.[L]	Y16	2	776.885	1552.763	1.02	7–19	Ch_MS/MS_-1
[K].DPSLVYEGIPM.[I]	-	2	610.797	1220.586	0.73	29–39	Ch_MS/MS_-2
[Y].LLVDLESK.[K]	-	2	458.770	916.534	0.81	50–57	Ch_MS/MS_-3

“-“, missed cleavage sites not retrieved; n.r., not reportedin Figure 2.

**Table 2:** Amino acid sequences and the molecular mass values of endoproteinase Arg-C (Arg-), chymotryptic (Ch-) and tryptic (T-) peptides as well as CNBr (CB-) fragments used to assemble the amino acid sequence of quinoin from *Chenopodium quinoa*.

Peptide	Sequence Position	Experimental Molecular Mass	Theoretical Molecular Mass ^a^	Δ(Da)	Missed Cleavage at	Reported in Figure 2
Arg-C peptides						
1	1–24	2811.68	2811.47	0.21	-	Arg-1
2	25–41	1934.75	1934.00	0.75	-	Arg-2
chymotrypsin peptides						
1	40–49	1173.57	1173.59	0.02	-	Ch-1
2	50–64	1791.26	1790.99	0.27	-	Ch-2
3	84–91	857.48	857.47	0.01	-	Ch-3
4	93–103	2405.82	2405.31	0.51	-	Ch-4
trypsin peptides						
1	59–71	1505.68	1505.83	0.15	K60	T-1
2	72–86	1643.64	1643.84	0.20	K82	T-2
3	103–133	3489.78	3489.76	0.02	K104, K105	T-3
4	167–187	2438.91	2438.37	0.54	R181, K183	T-4
5	199–219	2311.03	2311.32	0.29	K211, R214, K218	T-5
6	220–244	3490.28	3487.87 ^b^	2.41	-	T-6
6′	220–244	3636.56	3635.99 ^c^	0.57	-	(T-6)
CNBr fragments						
1	148–153	678.36	678.35 ^d^	0.01	-	CB-1
2	154–174	2394.34	2394.30 ^d^	0.04	-	CB-2
3	248–254	793.46	793.00 ^d^	0.46	-	CB-3

^a^, the theoretical molecular masses were obtained from the protein synthesis inhibitor PD-S2-like (A.C.: XP_021750597.1) retrieved in the *C. quinoa* genome. ^b^, the theoretical molecular mass was obtained considering the molecular masses of peptide (2772.19 Da) plus the glycan chain [(Hex)_1_(HexNAc)_2_(Deoxyhexose)_1_] (714.67 Da). ^c^, the theoretical molecular mass was obtained considering the molecular masses of peptide (2772.19 Da) plus glycan chain [(Hex)_2_ (HexNAc)_2_ (Pent)_1_] (862.79 Da). ^d^, [M + H^+^]^+^ experimental molecular mass values obtained by MALDI-TOF MS. The monoisotopic molecular masses were considered. The theoretical molecular weight was obtained considering the presence at the C-terminal of homoserine lactone, except for CB-3 for which homoserine was considered. “-”, missed cleavage sites not retrieved. Δ(Da), difference between theoretical and experimental molecular masses.

**Table 3:** MALDI-TOF-MS analysis of the peptide mixtures obtained by the trypsin digest of pre-quenoin-1 and pre-quinoin-2. The theoretical molecular masses were obtained from the quinoin sequence (see Figure 2a).

Theoretical Molecular Masses	Sequence Position	Experimental Molecular Masses ^a^				Missed Cleavage
		*Pre-Quinoin-1*	Δ(Da)	*Pre-Quinoin-2*	Δ(Da)	
790.41	245–251	790.72	0.31	790.73	0.32	-
813.64	150–156	813.66	0.02	813.68	0.04	-
1234.64	188–198	1234.53	0.11	1234.58	0.06	-
1250.67	134–144	1250.56	0.11	1250.60	0.07	-
1254.63	72–82	1254.51	0.12	1254.58	0.05	-
1444.77	199–211	1444.45	0.32	1444.53	0.24	-
1620.80	98–102	1620.32	0.48	1620.28	0.52	-
1643.84	72–86	1643.33	0.51	n.r.		K82
1664.80	145–158	1664.30	0.50	n.r.		K149, K156
1819.93	89–104	1819.31	0.62	n.r.		K102
2150.12	7–24	2149.27	0.85	2149.31	0.81	-
2278.27	163–182	2277.31	0.96	n.r.		R166, R180
2311.32	199–219	2311.29	0.03	n.r.		K211, R214, K218
2438.37	167–187	2439.34	0.97	2439.11	0.74	R180, K182

n.r.: not retrieved. ^a^, [M + H^+^]^+^ experimental molecular mass values obtained by MALDI-TOF MS. The monoisotopic molecular masses have been considered. “-”, missed cleavage sites not retrieved. Δ(Da), difference between theoretical and experimental molecular masses.

**Table 4:** Antimicrobial activity of quinoin from the seeds of *Chenopodium quinoa*. The assays were carried out as reported in the ‘Material and Methods’ section and the values shown were obtained from three independent experiments.

Microbe	Zone of Inhibition (mm ± SD)		
	Protein Amount		
	0.2 µg	0.5 µg	1.0 µg
Bacteria			
*Pseudomonas syringae* pv *phaseolicola*	2.5 ± 0.3	3.4 ± 0.5	4.0 ± 0.5
*Pseudomonas syringae* pv *actinidiae*	1.5 ± 0.3	1.8 ± 0.3	2.0 ± 0.4
*Salmonella enterica*	0.0	0.0	0.0
*Staphylococcus aureus*	0.0	0.0	0.0
Fungi			
*Cryponectria parasitica* strain E4	0.0	0.0	0.0
*Cryponectria parasitica* strain E13	0.0	0.0	0.0

## Data Availability

Raw data can be provided by the corresponding author on request.

## Supplementary Materials

The following are available online at https://www.mdpi.com/article/10.3390/ijms22168964/s1.
