# Supplementary material for: The Structural Characterization and Antipathogenic Activities of Quinoin, a Type 1 Ribosome-Inactivating Protein from Quinoa Seeds
Source: Int J Mol Sci. 2021 Aug 20;22(16):8964. doi: 10.3390/ijms22168964 (PMC8396469; doi:10.3390/ijms22168964)

SUPPORTING INFORMATION

The Structural Characterization and Antipathogenic Activities of Quinoin, a Type 1 Ribosome-Inactivating Protein from Quinoa Seeds

Sara Ragucci [a], Daniela Bulgari [b], Nicola Landi [a], Rosita Russo [a], Angela Clemente [a], Mariangela Valletta [a], Angela Chambery [a], Emanuela Gobbi [b], Franco Faoro [c], Antimo Di Maro [a, *]

[a] Department of Environmental, Biological and Pharmaceutical Sciences and Technologies (DiSTABiF), University of Campania 'Luigi Vanvitelli', Via Vivaldi 43, I-81100 Caserta, Italy

[b] Agri-food and Environmental Microbiology Platform (PiMiAA), Department of Molecular and Translational Medicine, University of Brescia, Viale Europa, 11, 25123, Brescia, Italy

[c] Department of Agricultural and Environmental Sciences, University of Milan, Via Celoria 2, 20133 Milan, Italy

* Corresponding author: e-mail: antimo.dimaro@unicampania.it (ADM); Tel: +39 0823 274409, ORCID ID: http://orcid.org/0000-0002-9595-9665.

Fig. S1. Purification of RIPs from *C. quinoa* seeds. (a), Elution profile from the CM-Sepharose chromatography showing three main active peaks. (b) and (c), re-chromatography FPLC elution profiles of peak 1 and peak 2, respectively, on an AKTA Purifier System from cation exchange chromatography using a Source 15S PE 4.6/100 column. Experimental conditions are described in the Material and Methods.

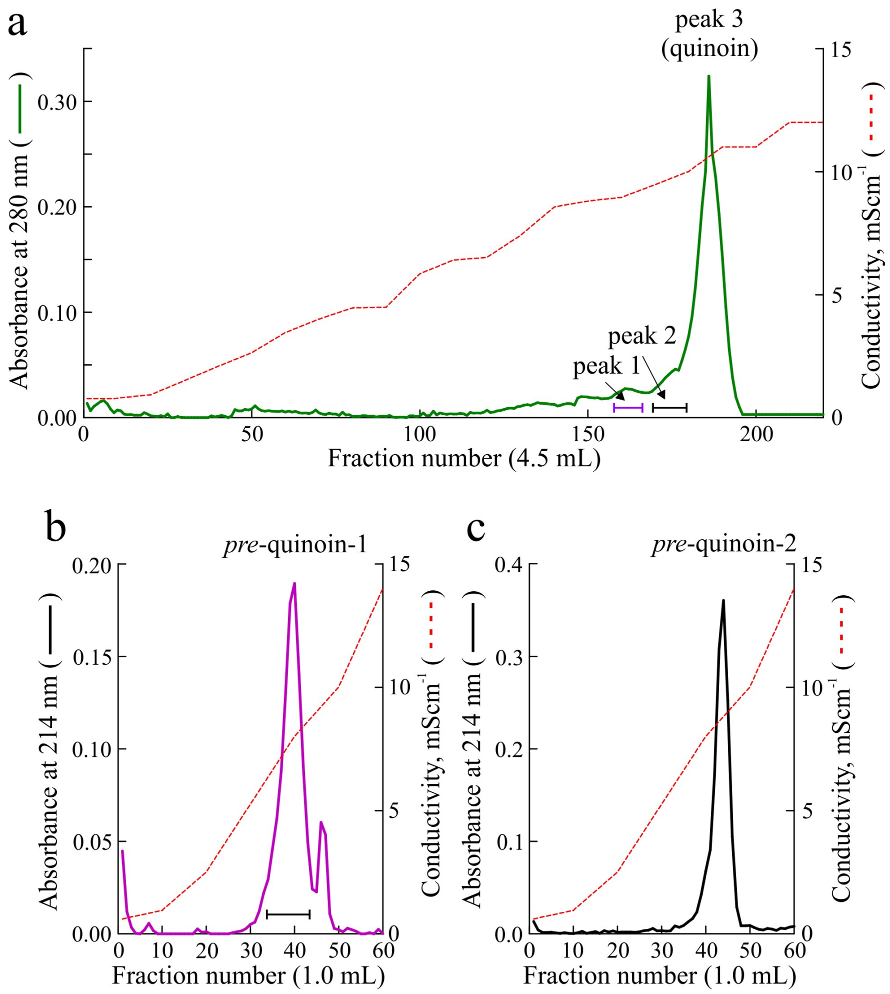

Table S1. Accesson numbers, species and common names of ribosome inactivating proteins sequences used in this work for primary structure analyses.

	Accession number	Species	name	Identity/Similarity (%) compared to quinoin	Note
1	Q03464	*Phytolacca americana* L.	PAP-alpha	40.94/48.81	
2	P34967	*Phytolacca dioica* L.	PD-S2	38.97/47.24	
3	P84854	*Phytolacca dioica* L.	PD-L3/4	39.37/48.42	
4	P84853	*Phytolacca dioica* L.	PD-L1/2	38.58/46.06	
5	P23339	*Phytolacca americana* L.	PAP-S	38.58/46.45	
6	P10297	*Phytolacca americana* L.	PAP-I	36.61/46.06	
7	Q6EH50	*Phytolacca heterotepala* L.	heterotepalin-4	35.82/44.48	
8	Q40772	*Phytolacca americana* L.	PAP-II	35.82/42.51	
9	P85208	*Phytolacca dioica* L.	dioicin-2	33.07/42.12	
10	P20656	*Saponaria officinalis* L.	saporin-6	29.24/35.17	
11	P27559	*Saponaria officinalis* L.	saporin-2	27.55/33.85	
12	Q41391	*Saponaria officinalis* L.	saporin-7	28.85/34.78	
13	P24476	*Dianthus caryophyllus*	dianthin-30	28.74/36.61	
14	Q41389	*Saponaria officinalis* L.	saporin-5	27.66/33.59	
15	Q7M1Z2	*Saponaria officinalis* L.	saporin-9	26.87/33.99	
16	P85101	*Silene chalcedonica* L.	lychnin	27.77/34.18	
17	P27560	*Saponaria officinalis* L.	saporin-3	26.69/32.20	
18	P21326	*Mirabilis jalapa* L.	MAP-S	24.00/31.20	
19	P02879	*Ricinus communis* L.	ricin	24.01/33.85	A-chain
20	P06750	*Ricinus communis* L.	agglutinin	23.62/32.28	A-chain

Fig. S2. Alignment of the amino acid sequences of the six proteins retrieved in *C. quinoa* genome classified as 'protein synthesis inhibitor like'. A.C.: XP_021750597.1, protein synthesis inhibitor PD-S2-like; A.C.: XP_021750694.1, ribosome inactivating protein PD-L3/PD-L4-like; A.C.: XP_021737780.1, protein synthesis inhibitor PD-S2-like_(2); A.C.: XP_021726635.1, ribosome inactivating protein PD-L3/PD-L4-like; A.C.: XP_021735486.1, antiviral protein MAP-like; A.C.: XP_021750669.1. Identical residues (*), conserved substitutions (:), and semi-conserved substitutions (.) are reported.

```
XP_021750597.1      ------------------------------------------------------------
XP_021750694.1      ------------------------------------------------------------
XP_021737780.1      ------------------------------------------------------------
XP_021726635.1      ------------------------------------------------------------
XP_021735486.1      ------------------------------------------------------------
XP_021750669.1      MRAFVVVAISIWLIVLEPTFAASDLSFDATSPTTNTYSKLLSDLREWLADPTLRYGNTNV


XP_021750597.1      ------------------------------------------------------------
XP_021750694.1      ------------------------------------------------------------
XP_021737780.1      ------------------------------------------------------------
XP_021726635.1      ------------------------------------------------------------
XP_021735486.1      ------------------------------------------------------------
XP_021750669.1      PVMAATFKKRKYLKGSEGGTITIGKCLKSKTYLISNKMLLLLMMMLWMYCGDGTNYLSKV


XP_021750597.1      ---------MQQENKKAWLVLTIAIWVVLQQVNAADVT---FKLEPKPTQNTYNTFLQSI
XP_021750694.1      -------MNGIQTIMRAFVVVAISTWLVILKPTFAASD-LSFDATN-PAATTYSKLLSDL
XP_021737780.1      -----MVGIKKFKIVSAYVVVAVAVWMVLEPTLAASDF--TFDIEG-ATTDTYSKLLTDV
XP_021726635.1      --------MVGIKVMGACVVVAIAVWMVLEPTLAASDIRISFDVEG-ATTATYSQLLNDV
XP_021735486.1      --MKLLAAPLSVIIWWCMMVMMAVVPPLTTAQDRETLSYSTVTFELTNGVTGYSSFLTRL
XP_021750669.1      NKGSMKLLAAPISVIWWCMMMMAVVPPLATAQDGETLAYSTVTFELTNGVTGYQSFLTRL
:::      :             .          *. :*  :

XP_021750597.1      RSQTKDPSLVYEG---IPMIRPPTNPDTYLLVDLESKKDKNEIFVTLALSRNDLYVVAFA
XP_021750694.1      RNLVKDPKLKYGG-TNVPVMAAT-FKKKYILVDLKGSEGGTITIALNLNN--DIYVVGYL
XP_021737780.1      RNKVKDDKIVYGGRKDIPVMAAPSAAKKYLFVDFTASEGRTITIAVNLSNDFNLYVIAYL
XP_021726635.1      RNKVKDKNIVYGGLKDLPVMAAP--SDNYLYVDLTASQKRTITLAVNLNKGFSLYVIAYL
XP_021735486.1      RNRVEAP---TRACTLQSTRNPPLTGAEYVLVDLKISNTQWVTLGIDAK---DLYVWAYQ
XP_021750669.1      RNQVESP---TRACTLQSTRNPPLTGAEYVLVDLKISNTQWVTLGIDAK---DLYVWAYQ
*. .:       .    .   ..     *: **:  .:     :        .:** .:

XP_021750597.1      D--KFGGKVRGHFFSNLNIDTIDKAKKVFPEVQVFINITYGESYSQIESNAGTN-RLSFP
XP_021750694.1      D--KLNGNFRSHIFKDAPSDAKTDLFPEATGKN-RLTINYKSSYADIESNAGVSSRARVG
XP_021737780.1      D--KINGNFRSHIFKDLPPDVQEGLFRDATGAANRLIMNYGSSYAEVEKKAGS--RTKLG
XP_021726635.1      D--KINGNFRSHIFKDLPNNVKDNLFPEAKGKANRLIMNYKSDYASIESKAGS--RNKLG
XP_021735486.1      DNVKYNGKYRANFLSDAPQAAKDRLFPGSTKR----TTRFGGNYNSLEPAAGTT-RKNLV
XP_021750669.1      DNVKYNGKYRATFLNDAPQAAKDRLFPGSTQR----TTRFGGNYNSLEPAAGTT-RKNLV
*  * .*: *. ::.:    .                  :  .* .:*  **   *  .

XP_021750597.1      LGFDNLKTYMEKVYGMDTKAKDYSKTEARFLLIAIQMVAEAARFKYIQGRAIVTTNPNN-
XP_021750694.1      LGVKPLNKFINDVYGKALVVKN----EAQFMLVVIQMVSEATRFKYIESMILEKFADSY-
XP_021737780.1      LGKKPLSKFFDKVYGKLVDNEN----EAKFMLIVMQMLSEATRFAYIEQMIVQKFEKEIN
XP_021726635.1      LGKQPLNIFFNKVYGKPVDDKN----QAKFMLVVMQMVSEATRFAYIENMIVQKFEQDIN
XP_021735486.1      LGIQNLDGAIRNVYNKQESELNKGTNEARFFLIAIQMVPEAARFKFMEQAIVRGDNSDS-
XP_021750669.1      LGIQNLDGAIRKVYNKQESELNKGTDEARFFLIAIQMVPEAARFRFMEQAIVRGDNSDS-
** . *.  : .**.      :    :*:*:*:.:**:.**:** :::   :     .

XP_021750597.1      ------YKILSLENNWGAISKGIRNAVKKV-----INPALILQYPNGTTWTVTQVSDIKN
XP_021750694.1      ---NPDPKAMRLEIRWSKISKGIKDSKKGV-----ISPELDLKDVNDKDWKVTKVEDIVN
XP_021737780.1      ECYKPDLKTIELEKSWQKITVGIKNSNGGV-----ICPPLKLQDPTGNPWIVSSVNEIAG
XP_021726635.1      ECYKPDFKMIELEKSWQKITVGIKNSKGGE-----IKPQLDLRGPNNEPWPVTRVDQIAK
XP_021735486.1      ----FKKKMVAFQNDWDPISQAIHKAEAATPKCVTITPTLIISN-VDYRQEVNRVDEIKN
XP_021750669.1      ----FKKKMVAFQNDWDPISQAIHKAEAATPKCVTITPTLIISN-IDYRQEVNRVDEIKN
* : ::  *  *: .*:.:         * * * :    .    *. *.:*

XP_021750597.1      DMGLLKYVM------------------------------------------
XP_021750694.1      DMALLKNFGSSSQVSVFTKLKLFMTKFLITNIGEDQAADL-----------
XP_021737780.1      DMGLLKYEGPPTTANNRVSLALVVNKLQTHYNQFISFF-------------
XP_021726635.1      DMGLLKYEGATKTANKGVYLGPFINKVQRHFNQFLSFLKANNGNDDDEAEQ
XP_021735486.1      DMGLLKYKSSTLSVIGSSILDDDI---------------------------
XP_021750669.1      DMGLLKYKSSTLSIIGSSILDDDI---------------------------
                    **.***
```

Fig. S3. Sequence alignment of ribosome inactivating proteins performed using the ClustalW tool (https://embnet.vital-it.ch/software/ClustalW.html) with default parameters. Asterisk, colon and dot are identical, conserved and semi-conserved amino acid residues, respectively. For protein information see **Table S1**. Cysteinyl residues are highlighted in red.

```
                          10        20        30        40        50        60        70        80
                          .         .         .         .         .         .         .         .

quinoin          -----ADVTFKLEPKPTQNTYNTFLQSIRSQTKDPSLVYEGIPMIRPPTNPDTYLLVDLESKKDK-NEIFVTLALSRNDL
PAP-alpha        ----INTITFDVG-NATINKYATFMKSIHNQAKDPTLKCYGIPMLPNTNLTPKYLLVTLQDSS----LKTITLMLKRNNL
PD-S2            ----VSTITFDVG-SATISKYTTFLESLRNQAKDPSLKCYGIPMLPNTNPNPKYLLVELNAKLSSGEVKSITLMLRRHNL
PD-L3/4          ----VNTITFDVG-NATINKYATFMESLRNEAKDPTLKCYGIPMLPDSNLTPKYVLVKLQDAS----SKTITLMLRRNNL
PD-L1/2          ----INTITYDAG-NTTINKYATFMESLRNEAKDPSLQCYGIPMLPNNSSTIKYLLVKLQGAS----QKTITLMLRRNNL
PAP-S            ----INTITFDAG-NATINKYATFMESLRNEAKDPSLKCYGIPMLPNTNSTIKYLLVKLQGAS----LKTITLMLRRNNL
PAP-I            ----VNTIIYNVG-STTISKYATFLNDLRNEAKDPSLKCYGIPMLPNTNTNPKYVLVELQGSNK----KTITLMLRRNNL
heterotepalin-4  ----VNTIIYNVG-STTISKYATFLDDLRNEAKDPNLKCYGIPMLPNTNSNPKYVLVELQGSNK----KTITLMLRRNNL
PAP-II           ------NIVFDVE-NATPETYSNFLTSLREAVKDKKLTCHGMIMATTLTEQPKYVLVDLKFGSG-----TFTLAIRRGNL
dioicin-2        ------NIVFDVE-NATPETYSSFLTSLREAVKDKKSTCHGMIMATTTTELPKYVLVDLKLGSEK-DAKTFTLAIRRGNL
saporin-6        -----VTSITLDLVNPTAGQYSSFVDKIRNNVKDPNLKYGGTDIAVIGPP-SKEKFLRINFQSS---RGTVSLGLKRDNL
saporin-2        -----VTSITLDLVNPTAGQYSSFVDKIRNNVKDPNLKYGGTDIAVIGPP-SKDKFLRINFQSS---RGTVSLGLKRDNL
saporin-7        -----VTSITLDLVNPTAGQYSSFVDKIRNNVKDPNLKYGGTDIAVIGPP-SKEKFLRINFQSS---RGTVSLGLKRDNL
dianthin-30      -----ATAYTLNLANPSASQYSSFLDQIRNNVRDTSLIYGGTDVAVIGAPSTTDKFLRLNFQGP---RGTVSLGLRRENL
saporin-5        -----VTSITLDLVNPTAGQYSSFVDKIRNNVKDPNLKYGGTDIAVIGPP-SKEKFLRINFQSS---RGTVSLGLKRDNL
saporin-9        -----VTSITLDLVNPTAGQYSSFVDKIRNNVKDPNLKYGGTDIAVIGPP-SKDKFLRINFQSS---RGTVSLGLKRDNL
lychnin          --------RPSWTVDSDSAKYSSFLDSLREEFGRGTPKVCNIPVTKKAN----NDKFVLVNLVLPFNRNTITLAFRASDA
saporin-3        --------------------------------DPNLKYGGTDIAVIGPP-SRDKFLRLNFQSS---RGTVSLGLKRENL
MAP-S            ------APTLETIASLDLNNPTTYLSFITNIRTKVADKTEQCTIQKISKTFTQRYSYIDLIVSS---TQKITLAIDMADL
ricin            IFPKQYPIINFTTAGATVQSYTNFIRAVRGRLTTGADVRHEIPVLPNRVGLPINQRFILVELSN-HAELSVTLALDVTNA
agglutinin       IFPKQYPIINFTTADATVESYTNFIRAVRSHLTTGADVRHEIPVLPNRVGLPISQRFILVELSN-HAELSVTLALDVTNA
                                                            :                          .:* :   :

                          90        100       110       120       130       140       150       160
                          .         .         .         .         .         .         .         .

quinoin          YVVAFADKFGG--KVRGHFFSNLN--IDTIDKAKKVFPEVQVF----INITYGESYSQIES----NAG-TNRLSFPLGFD
PAP-alpha        YVMGYADTYNG--KCRYHIFKDISNTTERNDVMTTLCPNPSSR--VGKNINYDSSYPALEK----KVGR-PRSQVQLGIQ
PD-S2            YVMGYSDPYDN--KCRYHIFKDISSTTERKDVETTLCPNASSR--VSKNISYDSSYPALEN----KAGR-SRSQVQLGIQ
PD-L3/4          YVMGYSDLYNG--KCRYHIFNDISS-TESTDVENTLCPNSNSR--EKKAINYNSQYSTLQN----KAGVSSRSQVQLGIQ
PD-L1/2          YVMGYSDPFNG--NCRYHIFNDITG-TERTNVENTLCSSSSSR--DAKPINYNSLYSTLEK----KAEVNSRSQVQLGIQ
PAP-S            YVMGYSDPYDN--KCRYHIFNDIKG-TEYSDVENTLCPSSNPR--VAKPINYNGLYPTLEK----KAGVTSRNEVQLGIQ
PAP-I            YVMGYSDPFETN-KCRYHIFNDISG-TERQDVETTLCPNANSR--VSKNINFDSRYPTLES----KAGVKSRSQVQLGIQ
heterotepalin-4  YVMGYSDPFDTS-KCRYHIFNDISG-TERQDVETTLCPNSNSR--VSKNINYDSRYPTLES----KVGVKSRSQVQLGIQ
PAP-II           YLEGYSDIYNG--KCRYRIFKDSES-----DAQETVCPGDKSKPGTQNNIPYEKSYKGMES----KGGA--RTKLGLGKI
dioicin-2        YLEGYSDIYNE--KCRYRIFEDSES-----DAQQTVCPGDLTLPGSQNKIPYKKSYQSMES----KGGD--RTKLGLGQI
saporin-6        YVVAYLAMDNTNVNRAYYFRSEITS-----AESTALFPEATTAN--QKALEYTEDYQSIEKNAQITQGDQSRKELGLGID
saporin-2        YVVAYLAMDNTNVNRAYYFKSEITS-----AELTALFPEATTAN--QKALEYTEDYQSIEKNAQITQGDKSRKELGLGID
saporin-7        YVVAYLAMDNTNVNRAYYFRSEITS-----AELTALFPEATTAN--QKALEYTEDYQSIEKNAQITQGDKSRKELGLGID
dianthin-30      YVVAYLAMDNANVNRAYYFKNQITS-----AELTALFPEVVVAN--QKQLEYGEDYQAIEKNAKITTGDQSRKELGLGIN
saporin-5        YVVAYLAMDNTNVNRAYYFRSEITS-----AELTALFPEATTAN--QKALEYTEDYQSIEKNAQITQGDKSRKELGLGID
saporin-9        YVVAYLAMDNTNVNRAYYFRSEITS-----AELTALFPEATAAN--HKALEYTEDYHSIEKNAQITEGDKSRKELGLGIN
lychnin          YLVGFQDRDSKTNKLRANFFSDEYR-----ALSGKYKSIFTDAEVLAPALPCASTYTDLQN----KAGV-SREKLSLGVS
saporin-3        YVVAYLAMDNANVNRAYYFGTEITS-----AELTTLLPEATVAN--QKALEYTEDYQSIEKNAKITEGDKTRKELGLGIN
MAP-S            YVLGYSDIAN--NKGRAFFFKDVTE-----AVANNFFPGATGTNR--IKLTFTGSYGDLEK----NGG--LRKDNPLGIF
ricin            YVVGYRAGNS------AYFFHPDNQ-----EDAEAITHLFTDVQN-RYTFAFGGNYDRLEQ----LAGN-LRENIELGNG
agglutinin       YVVGCRAGNS------AYFFHPDNQ-----EDAEAITHLFTDVQN-SFTFAFGGNYDRLEQ----LGG--LRENIELGTG
                 *: .              :                              :     *  ::.          * .  **

                          170       180       190       200       210       220       230       240
                          .         .         .         .         .         .         .         .

quinoin          NLKTYMEKVYGMDTKAKD-YSKTEARFLLIAIQMVAEAARFKYIQGRAIVTTN-----PNNYKILSLENNWGAISKGIRN
PAP-alpha        ILNSGIGKIYGVDSFT----EKTEAEFLLVAIQMVSEAARFKYIENQVKTNFN--RAFYPNAKVLNLEESWGKISTAIHN
PD-S2            ILNSDIGKISGVKSFT----DKTEAEFLLVAIQMVSEATRFKYIENQVKTNFN--RAFYPNAKVLNLEETWGKISMAIHG
PD-L3/4          ILNSDIGKISGVSTFT----DKTEAEFLLVAIQMVSEAARFKYIENQVKTNFN--RAFNPNPKVLSLEENWGKISLAIHN
PD-L1/2          ILSSDIGKISGQSSFT----DKTEAKFLLVAIQMVSEAARFKYIENQVKTNFN--RDFSPNDKILDLEENWGKISTAIHD
PAP-S            ILSSDIGKISGQGSFT----EKIEAKFLLVAIQMVSEAARFKYIENQVKTNFN--RDFSPNDKVLDLEENWGKISTAIHN
PAP-I            ILDSNIGKISGVMSFT----EKTEAEFLLVAIQMVSEAARFKYIENQVKTNFN--RAFNPNPKVLNLQETWGKISTAIHD
heterotepalin-4  ILDSDIGKISGVTSFS----EKTEAEFLLVAIQIS-EAARFKYIENQVKTNFN--RAFNPNPKVLNLEETWGKISTAIHD
PAP-II           TLKSRMGKIYGKDATDQKQYQKNEAEFLLIAVQMVTEASRFKYIENKVKAKFDDANGYQPDPKAISLEKNWDSVSKVIAK
dioicin-2        TLESRMNKIYGKDATDQKQFQKNEAEFLLIAVQMITEASRFKYIENKVKDSFDDAIGYKPDPKAISLETSWDKISNAIAK
saporin-6        LLSTSMEAVNKKARVVKD-----EARFLLIAIQMTAEAARFRYIQNLVIKNFP--NKFNSENKVIQFEVNWKKISTAIYG
saporin-2        LLLTFMEAVNKKARVVKN-----EARFLLIAIQMTAEVARFRYIQNLVTKNFP--NKFDSDNKVIQFEVSWRKISTAIYG
saporin-7        LLLTSMEAVNKKARVVKN-----EARFLLIAIQMTAEAARFRYIQNLVIKNFP--NKFNSENKVIQFEVNWKKISTAIYG
dianthin-30      LLITMIDGVNKKVRVVKD-----EARFLLIAIQMTAEAARFRYIQNLVTKNFP--NKFDSENKVIQFQVSWSKISTAIFG
saporin-5        LLLTSMEAVNKKARVVKN-----EARFLLIAIQMTAEVARFRYIQNLVTKNFP--NKFDSDNKVIQFEVSWRKISTAIYG
saporin-9        LLSSTMDTVNKKVRVVKN-----EARFLLIAIQMTAEAVRFRYIQNLVTKNFP--NKFNSENKVIKFEVNWKKISTAIHG
lychnin          SLQTAFTAVYGKVFTG-----KNVAKFALISIQMVAEAARFKYIEDQVINRGMYSSFEAG-ARITLLENNWSKISEQYHK
saporin-3        LLSTLMDAVNKKARVVKN-----EARFLLIAIQMTAEAARFRYIQNLVTKNFP--NKFNSEDKVIQFQVNWSKISKAIYG
MAP-S            RLENSIVNIYGKAG-----DVKKQAKFFLLAIQMVSEAARFKYISDKIPS--EKYEEVTVDEYMTALENNWAKLSTAVYN
ricin            PLEEAISALYYYSTGGT--QLPTLARSFIICIQMISEAARFQYIEGEMRTRIRYNRRSAPDPSVITLENSWGRLSTAIQE
agglutinin       PLEDAISALYYYSTCGT--QIPTLARSFMVCIQMISEAARFQYIEGEMRTRIRYNRRSAPDPSVITLENSWGRLSTAIQE
                  *   :  :               *.  ::.:*:  *. **:**..                    :: .*   :*
```

```
                         250       260       270       280       290
                          •         •         •         •         •
quinoin          A---VKKVINPALILQYPNGTTWTVTQVSDIKNDMG-LLKYVM-----------
PAP-alpha        A---KNGALTSPLELKNANGSKWIVLRVDDIEPDVG-LLKYVNGTCQAT-----
PD-S2            A---KNGAFSKPLELQNADGSKWIVLRVDEIKPDVA-LLKYVSGSCQAT-----
PD-L3/4          A---KNGALTSPLELKNADDTKWIVLRVDEIKPDMG-LLNYVSGTCQTT-----
PD-L1/2          A---TNGALPKPLELKNADGTKWIVLRVDEIKPDMG-LLNYVNGTCQTT-----
PAP-S            S---KNGALPKPLELKNADGTKWIVLRVDEIKPDVG-LLNYVNGTCQAT-----
PAP-I            A---KNGVLPKPLELVDASGAKWIVLRVDEIKPDVA-LLNYVGGSCQTTY----
heterotepalin-4  A---KNGVLPKPLELVDASGAKWIVLRVDEIKPDVA-LLNYVSGSCQTT-----
PAP-II           VGTSGDSTVTLPGDLKDENNKPWTTATMNDLKNDIMALLTHVTCKVKSSMFPEI
dioicin-2        VNTPGNSIVTLPKGLLDENKKPWTTATMDELKNDIMGLLTHVTCKIK-------
saporin-6        D--AKNGVFNKDYDFGFGKVRQVKDLQMGLLMYLGKPK----------------
saporin-2        D--AKNGVFNKDYDFGFGKVRQVKDLQMGLLMYLGKPKSSNEANSTAYATTVL-
saporin-7        D--AKNGVFNKDYDFGFGKVRQVKDLQMGLLMYLGKPK----------------
dianthin-30      D--CKNGVFNKDYDFGFGKVRQAKDLQMGLLKYLGRPKSSSIEANSTDDTADVL
saporin-5        D--AKNGVFNKDYDFGFGKVRQVKDLQMGLLMYLGKPK----------------
saporin-9        D--AKNGVFNKDYDFGFGKVRLVKDLQMGLLMHLGKPK----------------
lychnin          S---CKLGGGQFTEEEMKLG----------------LLLYN-------------
saporin-3        D--AKNGVFNKDYDFGFGKVRQVKDLQMGLLMYLGTTPNNAADRYRAEL-----
MAP-S            S---KPSTTTATKCQLATSPVTISPWIFKTVEEIKLVMGLLKSS----------
ricin            S---NQGAFASPIQLQRRNGSKFSVYDVSILIPIIALMVYRCAPPPSSQF----
agglutinin       S---NQGAFASPIQLQRRNGSKFNVYDVSILIPIIALMVYRCAPPPSSQF----
```

Fig. S4. Western blot analysis of quinoin (q1 and q2, 100 and 50 ng, respectively) and PD-S2, PD-L1 and PD-L4 (.1 and .2, 100 and 50 ng, respectively). Purified proteins were separated by SDS-PAGE and subjected to immunoblot analysis using anti-quinoin or anti-PD-S2 (type 1 RIP from seeds of *P. dioica*) rabbit polyclonal antibody.

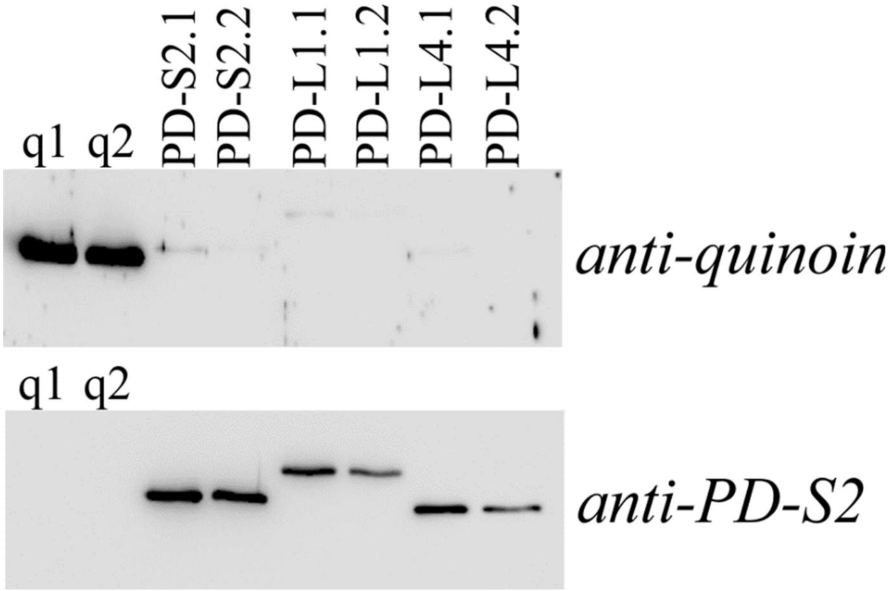

Fig. S5. Open Reading Frame (ORF) organization of quinoin derived from the genomic sequence NW_018745569.1.

```
atg caa caa gaa aat aag aag gca tgg ctt gtt ctc acg ata gct ata tgg gtc gtc ctt
 M   Q   Q   E   N   K   K   A   W   L   V   L   T   I   A   I   W   V   V   L
                     ▼ mature quinoin
caa cag gtt aat gca gct gat gta acc ttc aaa ctc gaa cca aaa cct aca cag aat act
 Q   Q   V   N   A   A   D   V   T   F   K   L   E   P   K   P   T   Q   N   T
tat aac acc ttt ctg caa agt ata cgc agc caa act aag gat cca agc tta gtg tat gaa
 Y   N   T   F   L   Q   S   I   R   S   Q   T   K   D   P   S   L   V   Y   E
gga atc cca atg atc cga cca cca acc aac cca gat aca tat ctt ttg gtt gac ctt gaa
 G   I   P   M   I   R   P   P   T   N   P   D   T   Y   L   L   V   D   L   E
tct aaa aag gat aag aat gag ata ttc gtt aca ctt gct tta agt aga aac gac ttg tat
 S   K   K   D   K   N   E   I   F   V   T   L   A   L   S   R   N   D   L   Y
gta gtg gcc ttt gct gat aaa ttt gga ggc aaa gtt cgc ggc cat ttc ttt tcc aat ctt
 V   V   A   F   A   D   K   F   G   G   K   V   R   G   H   F   F   S   N   L
aac att gac acc att gat aag gca aag aaa gtt ttt cca gaa gtt cag gtt ttc ata aat
 N   I   D   T   I   D   K   A   K   K   V   F   P   E   V   Q   V   F   I   N
ata acg tac ggg gaa agt tac agt caa atc gaa agc aat gct ggt aca aac cga tta agt
 I   T   Y   G   E   S   Y   S   Q   I   E   S   N   A   G   T   N   R   L   S
ttt ccg ttg ggg ttt gat aac ctt aaa act tac atg gaa aag gtc tat ggg atg gat act
 F   P   L   G   F   D   N   L   K   T   Y   M   E   K   V   Y   G   M   D   T
aag gcc aaa gat tat agc aaa act gaa gct cgg ttc cta ctc atc gcg att caa atg gtt
 K   A   K   D   Y   S   K   T   E   A   R   F   L   L   I   A   I   Q   M   V
gca gag gcc gca cgt ttc aag tac atc cag ggg aga gct atc gtt act aca aat cct aac
 A   E   A   A   R   F   K   Y   I   Q   G   R   A   I   V   T   T   N   P   N
aat tat aaa ata ctg tcg tta gag aac aac tgg gga gct att tcg aaa ggc att cgg aac
 N   Y   K   I   L   S   L   E   N   N   W   G   A   I   S   K   G   I   R   N
gct gtt aag aaa gtc atc aat cct gcg tta att tta caa tac cct aat ggt aca aca tgg
 A   V   K   K   V   I   N   P   A   L   I   L   Q   Y   P   N   G   T   T   W
aca gtt aca caa gtg agt gat ata aaa aat gat atg gga ctc ctt aag tat gtt atg taa
 T   V   T   Q   V   S   D   I   K   N   D   M   G   L   L   K   Y   V   M   -
```

Fig. S6. Representative images of bean cotyledonary leaves infected with TNV alone (a) or TNV mixed to 2.0 or 0.2 µg/mL of quinoin (b, c, respectively) on the adaxial leaf surface, or when inoculated separately from quinoin, the latter applied on the abaxial leaf surface (d).

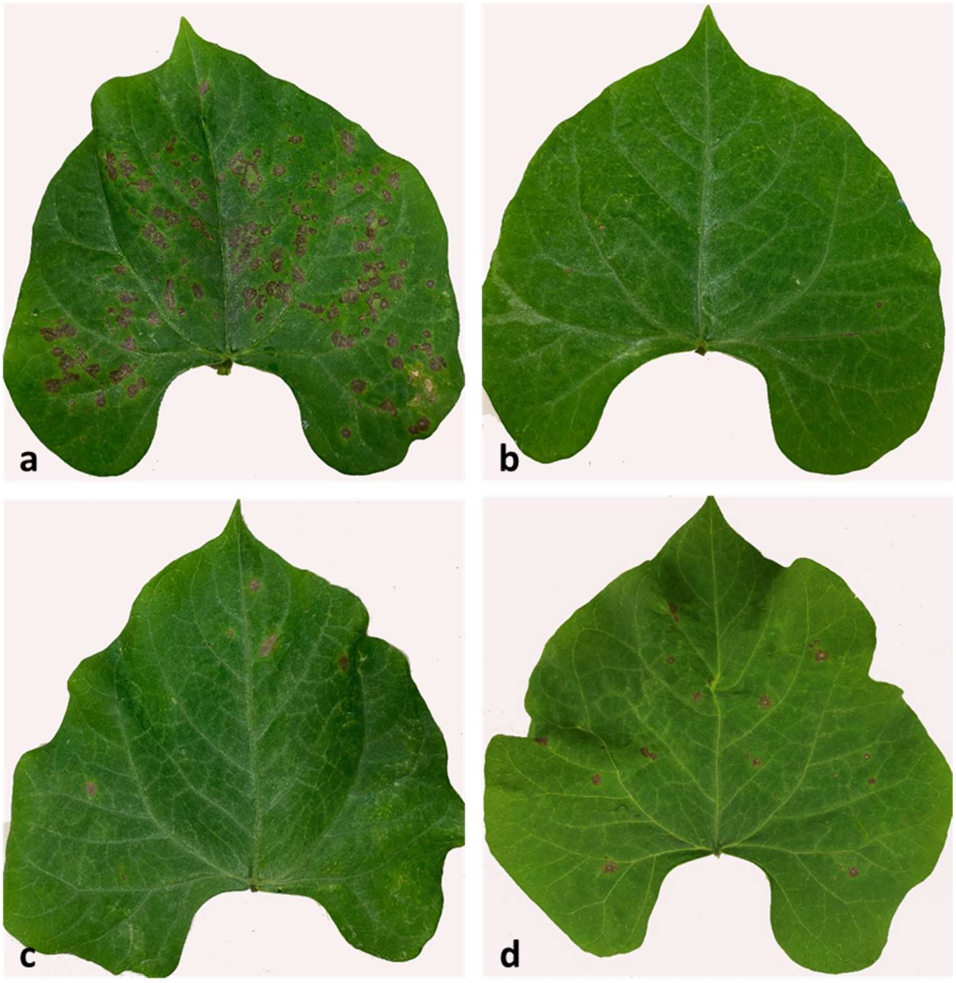

Fig. S7. Stereo microscopy analyses in order to evidence the antifungal effect of quinoin at different concentrations against the plant pathogenic fungi *Cryponectria parasitica* strain E4 (a) and *C. parasitica* strain E13 (b). Water, negative control.

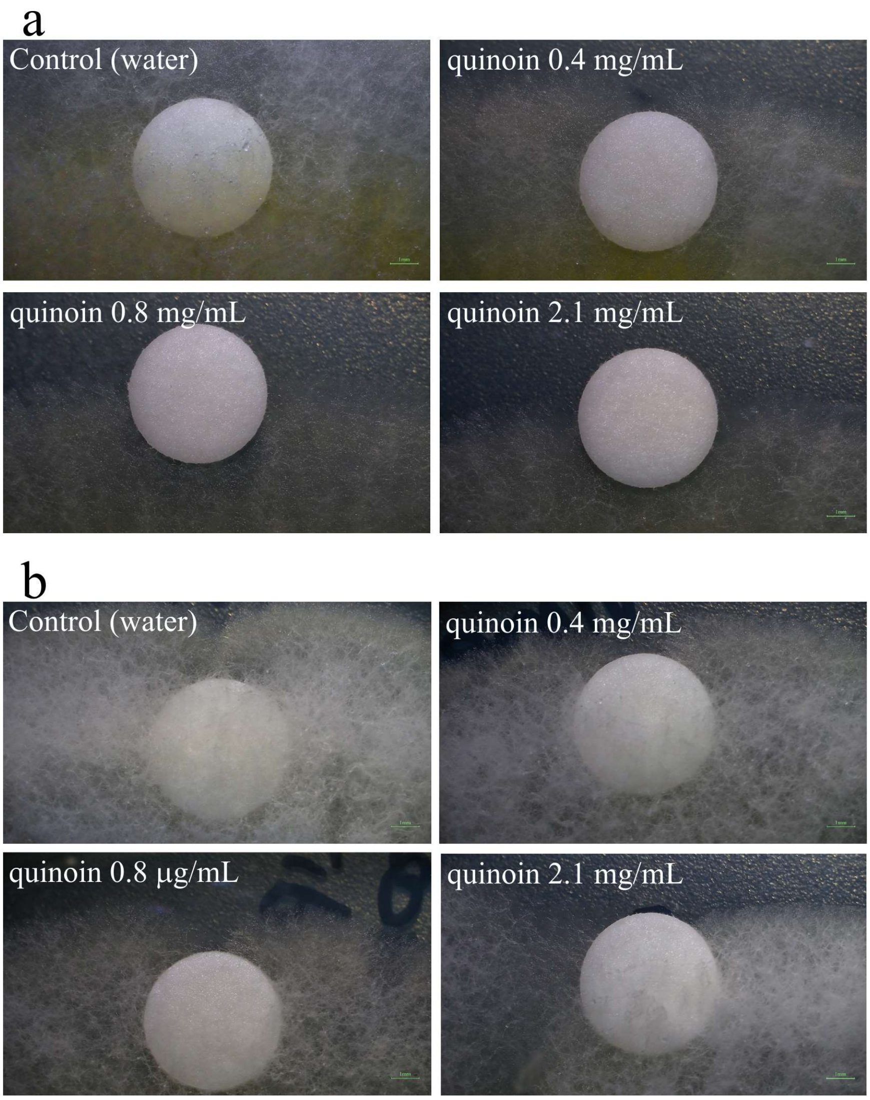

Supplement: Supplementary file 1 [file ijms-22-08964-s001.zip › ijms-1337013-supplementary.pdf]
